# Discovery and Rational Design of a Novel Bowman-Birk Related Protease Inhibitor

**DOI:** 10.3390/biom9070280

**Published:** 2019-07-14

**Authors:** Yuxi Miao, Guanzhu Chen, Xinping Xi, Chengbang Ma, Lei Wang, James F. Burrows, Jinao Duan, Mei Zhou, Tianbao Chen

**Affiliations:** 1 Natural Drug Discovery Group, School of Pharmacy, Queen’s University Belfast, Belfast, Northern Ireland BT7 1NN, UK; 2 Jiangsu Key Laboratory for Traditional Chinese Medicine (TCM) Formulae Research, Nanjing University of Chinese Medicine, Nanjing 210046, China

**Keywords:** amphibian Bowman-Birk inhibitor, Tat peptide, molecular cloning, antifungal, drug design, protease inhibitor

## Abstract

Anuran amphibian skin secretions are a rich source of peptides, many of which represent novel protease inhibitors and can potentially act as a source for protease inhibitor drug discovery. In this study, a novel bioactive Bowman-Birk type inhibitory hexadecapeptide of the Ranacyclin family from the defensive skin secretion of the Fukien gold-striped pond frog, *Pelophlax plancyi fukienesis*, was successfully isolated and identified, named PPF-BBI. The primary structure of the biosynthetic precursor was deduced from a cDNA sequence cloned from a skin-derived cDNA library, which contains a consensus motif representative of the Bowman-Birk type inhibitor. The peptide was chemically synthesized and displayed a potent inhibitory activity against trypsin (Ki of 0.17 µM), as well as an inhibitory activity against tryptase (Ki of 30.73 µM). A number of analogues of this peptide were produced by rational design. An analogue, which substituted the lysine (K) at the predicted P_1_ position with phenylalanine (F), exhibited a potent chymotrypsin inhibitory activity (Ki of 0.851 µM). Alternatively, a more potent protease inhibitory activity, as well as antimicrobial activity, was observed when P^16^ was replaced by lysine, forming K^16^-PPF-BBI. The addition of the cell-penetrating peptide Tat with a trypsin inhibitory loop resulted in a peptide with a selective inhibitory activity toward trypsin, as well as a strong antifungal activity. This peptide also inhibited the growth of two lung cancer cells, H460 and H157, demonstrating that the targeted modifications of this peptide could effectively and efficiently alter its bioactivity.

## 1. Introduction

Serine proteases are a widely studied group of proteins as they play various roles in healthy and diseased tissues. Serine protease inhibitors can also modulate a series of important biological processes, such as coagulation and inflammation, making them a focus for biomedical studies [1,2].

Plants are remarkable sources of the serine protease inhibitor, which can be grouped into at least 10 families. The Bowman-Birk family inhibitors (BBIs) are the best studied and most widely known among them. Identified in and isolated from soybean, they were the first to often be referred to as “classical BBI”. However, subsequently multiple BBIs have been isolated from plants such as legumes and Gramineae [3,4,5].

The skin of frogs is the main organ involved in their defense system, which manufactures diverse bioactive peptides that possess cytolytic pharmacological activities [6], and as a result it is also an excellent source of protease inhibitors. To date, many BBIs isolated from amphibians have been reported, such as peptide leucine arginine (pLR) [7], peptide tyrosine arginine (pYR) [8], the Bowman-Birk-like trypsin inhibitor from *Huia versabilis* (HV-BBI) [9], Hejiang trypsin inhibitor (HJTI) [10], the Bowman-Birk-type inhibitor from *Odorrana schmackeri* (OSTI) [11], *Hylarana erythraea* chymotrypsin inhibitor (HECI) [12] and *Pelophyla esculentus Bowman-Birk proteinase inhibitor* (PE-BBI) [13]. Generally, the BBI peptides from amphibians possess a highly-conserved 11-residue canonical disulfide loop, which is different from plant BBIs. The structure of this peptide follows the consensus sequence, CWTP_1_SXPPXPC, with P_1_ representing the inhibitory active site and X indicating that various amino acids are found in these positions. This disulfide-bridged loop is considered a trypsin inhibitory loop (TIL), which has a significant trypsin inhibitory activity [14].

Based on previous studies, these amphibian BBIs not only have potent protease inhibitory activities, but also exhibit an antimicrobial activity. Antimicrobial peptides (AMPs) are attractive alternatives to producing novel antibiotics. However, their susceptibility to proteases appreciably limits the potential applications of most AMPs. Therefore, a bifunctional peptide possessing both antimicrobial and protease inhibitory activities, with a low cytotoxicity, could represent an ideal template for future clinical use [15].

In this report, a novel peptide from the defensive skin secretion of the Fukien gold-striped pond frog, *Pelophlax plancyi fukienesis*, was successfully isolated and identified, named PPF-BBI. It was shown to possess a potent trypsin and tryptase inhibitory activity with a high specificity. Several analogues were created by rational design, and a P_1_ site F substituted analogue displayed a considerable and specific chymotrypsin inhibitory activity. A better antimicrobial activity was observed when P^16^ was replaced by Lys, and the addition of the cell-penetrating peptide Tat_48–56_ resulted in a peptide with a strong antifungal activity. Moreover, anti-proliferative effects on H157 and H460 were also observed.

## 2. Materials and Methods

### 2.1. Specimen Biodata and Secretion Harvesting

Specimens of the Fukien gold-striped pond frog, *Pelophelax plancyi fukienensis* (n = 3, snout-to-vent length 7 cm) were captured in Fuzhou City, Fujian Province, China. All frogs were adults, and skin secretion was obtained by a mild electrical stimulation on the dorsal skin surface of the frogs [16]. The secretion was collected by washing the skin using deionized water and was lyophilized after the liquid nitrogen freezing. The obtained secretion was stored at −20 °C. This study is approved by the Nanjing University of Chinese Medicine Ethical Review Board-Approval Code: SYXK (SU) 2018-0048.

### 2.2. Identification of Precursor-Encoding cDNA from the Skin Secretion

The precursor encoding cDNA from the skin secretion was obtained as described previously [17]. Briefly, the 3′-RACE reactions employed a nested universal (NUP) primer (supplied with the kit) and a sense primer (S: 5′-GCIYTIMGIGGITGYTGGACIAA-3′) that was complementary to the amino acid sequence, ALRGCWTK-, of PPF-BBI. The RACE reactions were purified and cloned using a pGEM-T vector system (Promega Corporation, Madison, WI, USA) and sequenced using an ABI 3100 automated sequencer. The nucleotide sequence of PPF-BBI has been deposited in the GenBank database under the accession number MK965542.

### 2.3. Isolation and Identification of PPF-BBI from Skin Secretion

The isolation and identification of the mature peptide from the skin secretion using the RP-HPLC and LC-MS analyses were performed as outlined previously [18]. A molecular mass analysis of the contents contained in the HPLC fraction was achieved by use of a matrix-assisted laser desorption ionization time-of-flight (MALDI-TOF) mass spectrometer (Voyager DE, Perseptive Biosystems, Framingham, MA, USA). The major peptide within this fraction was subjected to MS/MS fragmentation sequencing using an LCQ-Fleet mass spectrometer (Thermo Fisher Scientific, San Jose, CA, USA).

### 2.4. Peptide Design and Solid Phase Peptide Synthesis of PPF-BBI, F^8^-PPF-BBI, K^16^-PPF-BBI, Tat-loop, Tat and Trypsin Inhibitory Loop

There are three design strategies based on the obtained parent peptide, one to alter the inhibitory specificity, one to enhance the antimicrobial activity, and one to enhance the drug delivery and cell targeting. In the first case, since synthetic work on the BBI-like peptides has focused mostly on the P_1_ site, the lysine at the P_1_ site was replaced by a phenylalanine (F^8^-PPF-BBI; ALRGCWTFSIPPKPCP-NH_2_) which confers chymotrypsin inhibitory specificity. In the second case, the last amino acid residue, proline, was substituted with a lysine to give a positive charge and achieve a more structural similarity with the members of the Ranacyclin family, which have an antimicrobial activity (K^16^-PPF-BBI; ALRGCWTKSIPPKPCK-NH_2_). The trypsin inhibitory loop (TIL, CWTKSIPPKPC), derived from the amphibian Bowman-Birk-type protease inhibitor, is found to have a potent trypsin inhibitory activity. Thus, in the last case, a short cell-penetrating peptide Tat_48-56_ (RKKRRQRRR), which has been considered one of the most promising tools to improve the cellular delivery of therapeutic molecules [19,20,21], was added to the N-terminal of the TIL (Tat-loop; RKKRRQRRRCWTKSIPPKPC) by solid phase peptide synthesis. The Tat peptide and TIL were used in the antimicrobial assays for a comparison with the activity of Tat-loop. The TIL peptide was also involved in protease inhibitory assays to determine the influence on the inhibitory activity of the extended amino acid residues at both termini.

The novel-cloned cDNA-encoded peptide, wild-type PPF-BBI, and its analogues were synthesized by chemical synthesis using a Tribute peptide solid-phase synthesizer (Protein Technologies, Inc, Tucson, AZ, USA), as outlined previously [11]. The synthetic peptides were analyzed both by reverse phase HPLC and MALDI-TOF mass spectrometry to establish the degree of purity and the identity of the structure.

### 2.5. Trypsin, Chymotrypsin and Tryptase Inhibition Assay

The trypsin, chymotrypsin inhibition tests were performed as described previously [22]. 10 µL tryptase (1 mg/mL, Calbiochem, UK) was added to the wells of a micro-titer plate containing 180 µL substrate (Boc-Phe-Ser-Arg-NHMec, obtained from Bachem, UK) (50 µM) and 20 µL synthetic replicates (0.1–100 µM) in a tryptase buffer, pH7.6, containing 0.05 M Tris, 0.15 M NaCl, and 0.2% (*w*/*v*) polyethylene glycol 6000 (final volume 210 µL).

The rate of hydrolysis of the substrate was monitored by measuring the rate of increase of fluorescence due to the release of 7–amino–4–methylcoumarin (AMC) at 460 nm (excitation 360 nm) in a FLUOstar OPTIMA multi-well plate reader. The inhibition curves of the trypsin/chymotrypsin inhibition assay and tryptase inhibition assay were formed as outlined before [11,12].

### 2.6. Minimal Inhibitory Concentration (MIC) Assay and Minimal Batericidal Concentration (MBC) Assay

The MIC and MBC of the synthetic peptides were determined as previously described [17], using *S. aureus* (NCTC 10788), *E. coli* (NCTC 10418) and *C. albicans* (NCYC 1467), together with two species of resistant micro-organisms, methicillin-resistant *S. aureus* (MRSA) (ATCC 12493) and *P. aeruginosa* (ATCC 27853).

### 2.7. Membrane Permeability Assay

The membrane permeability assay was performed as described previously [20]. The peptides at concentrations of 1-fold MIC, 2-fold MIC and 4-fold MIC were mixed with a bacterial cell suspension. The membrane permeability rate was measured via the monitor of the fluorescent intensity of SYTOX Green Nucleic Acid Stain (Life Technologies, Glasgow, UK) by a Synergy HT plate reader with excitation at 485 nm and emission at 528 nm.

### 2.8. Secondary Structure Analysis through Circular Dichroism (CD)

The sample peptide solutions (50 μM) were prepared in a 1 mm high precision quartz cell (Hellma Analytics, Essex, UK) with 10 mM ammonium acetate and 50% TFE in 10 mM ammonium acetate buffer respectively. CD measurements were performed at 20 °C by a JASCO J-815 CD spectrometer (Jasco, Essex, UK) across the wavelength range of 190–250 nm. The scanning speed was 100 nm/min, the bandwidth was one nm, and the data pitch was 0.5 nm. The CD spectra were further analysed using the online software, BeStSel [23], and the proportion of different secondary structures were predicted.

### 2.9. MTT Assay

The MTT assay was carried out as described in a previous study [20], using a series of lung cancer cell lines, NCI-H157 (RRID: CVCL_0463), NCI-H460 (ATCC^®^ HTB-177™), H838 (ATCC^®^ CRL-5844™), and H23 (ATCC^®^ CRL-5800™), as well as other cancer cell lines: HT-29 (ATCC^®^ HTB-38™), PC-3 (ATCC^®^ CRL-1435™), U251MG (ECACC-09063001), and the normal human dermal microvascular endothelium cell line HMEC-1 (ATCC^®^ CRL-3243™). The anti-metabolite 5-fluorouracil (5-FU) was utilized as the positive control group.

### 2.10. Haemolysis Test

The haemolytic activity of each peptide was measured by incubating a range of final peptide concentrations from 512 to 1 µM in a two-fold dilution in a 2% suspension of horse erythrocytes, as described in a previous study [20].

### 2.11. Statitical Analysis

The data of all the bioactive assays were statistical analyzed using Prism 6 (GraphPad Prism Software, GraphPad, San Diego, CA. USA). The data points represent the average of three independent experiments with the error bars representing the standard error of the mean (SEM).

## 3. Results

### 3.1. Identification and Structural Determination of PPF-BBI

**Figure 1:**
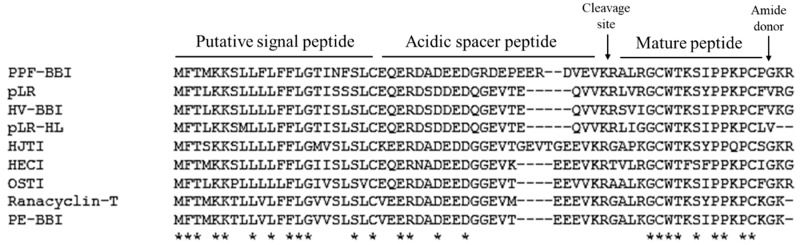
Multiple sequence alignment test results from Clustal Omega. Fully conserved residue indicated by asterisks.

From a skin-derived cDNA library, a cDNA encoding a biosynthetic precursor of PPF-BBI was consistently and repeatedly cloned (Figure S1). The crude skin secretion of *Pelophlax plancyi fukienesis* was analysed by LC-MS. The retention time of the fragmentation indicated in Figure S2 showed a corresponding molecular weight (Figure S3A) to the prediction for PPF-BBI according to the putative peptide from the cloned cDNA. The elution fraction was analysed by an electrospray mass spectrometer, and the primary structure sequence was confirmed (Figure S3B,C). The open-reading frame consisted of 65 amino acid residues. The alignment of this peptide with other members of the Ranacyclin family of Bowman-Birk-type protease inhibitors indicates that it is structurally related as its sequence shows a high degree of conservation, as well as including a typical inhibition loop (Figure 1), which started with a 22-residue putative signal peptide at the N-terminus. After the acidic spacer of 24-amino acids, the deduced mature peptide of 16 residues at the C-terminus is present in a single copy (Figure 1). The sequence was preceded by two consecutive basic amino acids, Lys-Arg (KR), representing a typical processing site for endoproteolytic cleavage, and was immediately followed by a glycine residue amide donor.

### 3.2. Peptide Design

Three analogues were designed based on the natural peptide PPF-BBI (Table 1). Briefly, a substitution of phenylalanine at the P_1_ site, F^8^-PPF-BBI, was aimed to produce a chymotrypsin inhibitory peptide. The substitution of a lysine at the position 16 of the native peptide (K^16^-PPF-BBI) enhanced the net positive charge, which might improve the ability to interact with the cell membrane. The Tat sequence was added at the N-terminus of the typical 11-mer trypsin inhibitory loop structure to increase the membrane penetration effect. All the analogues were chemically synthesized, purified by RP-HPLC and analysed by MALDI-TOF.

### 3.3. Synthesis and Secondary Structure Analysis of PPF-BBI and its Analogues

**Figure 2:**
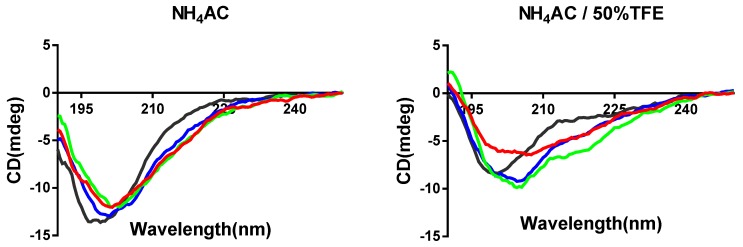
Secondary structures of PPF-BBI and the analogues. The CD spectra of peptides were measured in their free form (aqueous 10 mM NH_4_AC buffer) and membrane-mimic 10 mM NH_4_AC/50% TFE buffer, respectively (PPF-BBI, red; F^8^-PPF-BBI, green; K^16^-PPF-BBI, blue; Tat-loop, black).

PPF-BBI and the analogue peptides were successfully synthesized, impurities were removed by HPLC, and their identity was confirmed by MALDI-TOF. The secondary structures of all peptides were determined by circular dichroism spectroscopy (Figure 2). PPF-BBI, F^8^-PPF-BBI and K^16^-PPF-BBI revealed a broad negative band with the minimum around 200 nm, typical of an unfolded peptide in equilibrium with a β-sheet structure [24], except that Tat-loop exhibited the negative band close to 197 nm, which is considered to be a random coil. With the presence of 50% TFE, which is a secondary structure promoting the reagent, the negative minimum of PPF-BBI shifted from 200–203 nm to 206–210 nm and displayed a broad negative band. Additionally, F^8^-PPF-BBI and K^16^-PPF-BBI displayed the same red shift trend of the negative minimum. Since the spectra displayed a broad minimum spanning the region 200–210 nm and did not show positive bands above 210 nm, this suggests that the conformation of peptides is likely to consist of a mixture of secondary structures of β-sheet structure and random coil, which is consistent with previous studies [11,24,25,26]. Furthermore, the Tat peptide possess a random coil structure, the presence of which would not increase the helicity of the peptide [26].

### 3.4. Trypsin, Chymotrypsin and Tryptase Inhibitory Activity of PPF-BBI and its Analogues

PPF-BBI and its analogues were tested for inhibitory activity against trypsin, chymotrypsin and tryptase, respectively. The progress curves for the hydrolysis of the fluorogenic substrate were used to estimate an initial rate (Vi) to generate the Morrison plots. All of the progress curves and corresponding Morrison plots in the presence of each peptide of different concentrations are shown in Figure S4. Among these, both wild type PPF-BBI and K^16^-PPF-BBI exhibited a potent trypsin inhibitory activity. However, K^16^-PPF-BBI had a more potent inhibitory effect against tryptase than the parent peptide did. In addition, F^8^-PPF-BBI only displayed a strong chymotrypsin inhibitory activity and lost the trypsin inhibitory activity. Interestingly, the trypsin inhibitory loop (TIL) and Tat-loop kept the trypsin inhibition activity but did not exhibit a tryptase inhibitory activity (Table 2).

### 3.5. Antimicrobial Activity

The antimicrobial activity of PPF and its analogues was tested against a representative set of microorganisms (Table 3). Both PPF-BBI and K^16^-PPF-BBI displayed a mild activity against the tested microorganisms, although K^16^-PPF-BBI exhibited a better bioactivity than the parent peptide against *S. aureus* and *C. albicans*. However, although its activity against the other microorganisms was similar to the parental peptide, Tat-loop showed a much more potent activity against *C. albicans*. The component parts of Tat-loop (separate Tat peptide and the TIL) were also tested, but had little activity on their own. To sum up, Tat-loop showed a potent activity against *C. albicans*, as well as exhibiting slightly more activity against MRSA and *P. aeruginosa*. K^16^-PPF-BBI exhibited the best activity against *S. aureus* and was slightly better than PPF-BBI against all of the others except *E. coli*.

### 3.6. Membrane Permeability

**Figure 3:**
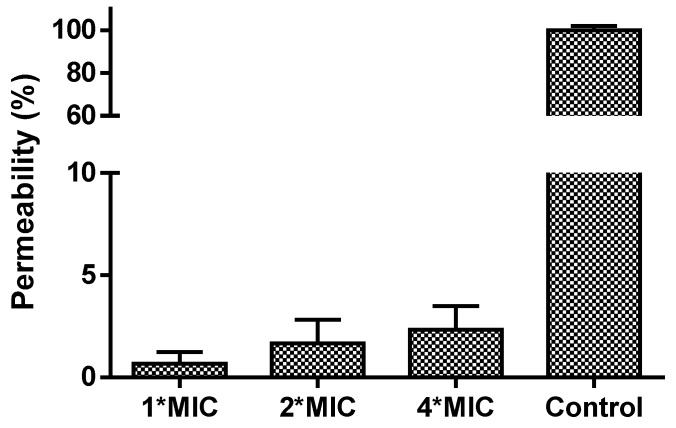
The cell permeability of *C. albicans* treated for 2 h by Tat-loop at 1-fold, 2-fold and 4-fold of MIC. The membrane permeabilized cells by 70% isopropanol were used as the positive control (100% permeability).

The MIC and MBC results indicated that only Tat-loop had a potent activity on any of the tested microorganisms. Therefore, it was tested to determine if its impact against *C. albicans* was due to it impacting its membrane permeability. However, Tat-loop did not cause any membrane permeabilization, even at high concentrations (4-fold of its MIC versus *C. albicans*, Figure 3).

### 3.7. Anti-Cancer and Haemolytic Activity

**Figure 4:**
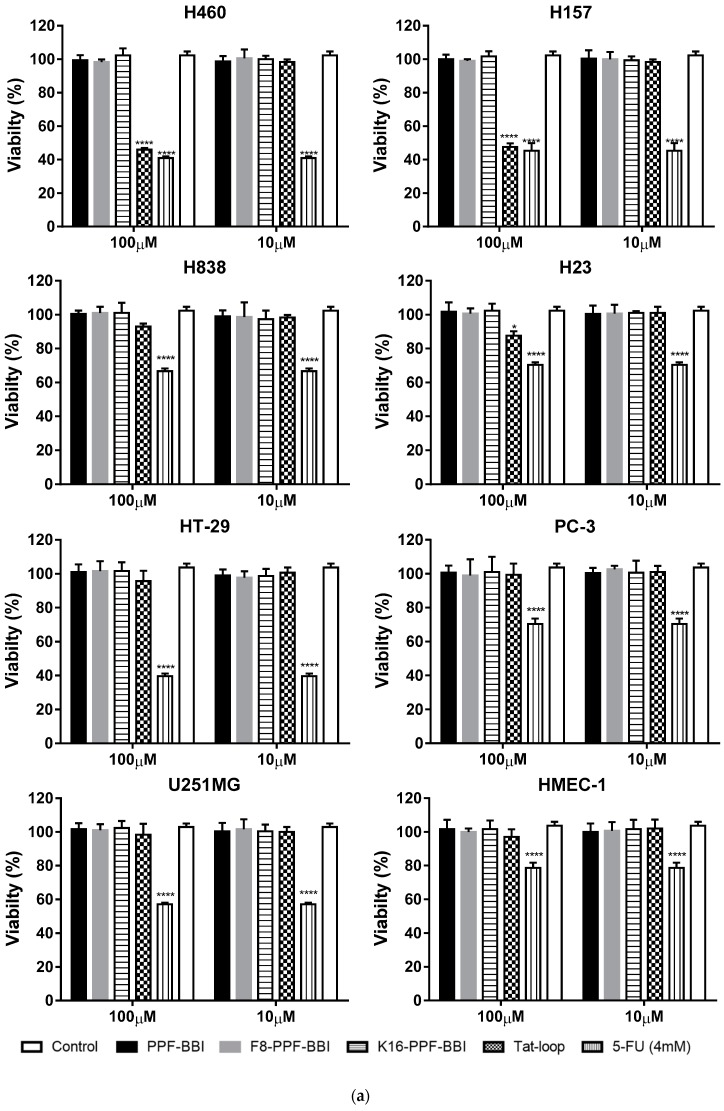

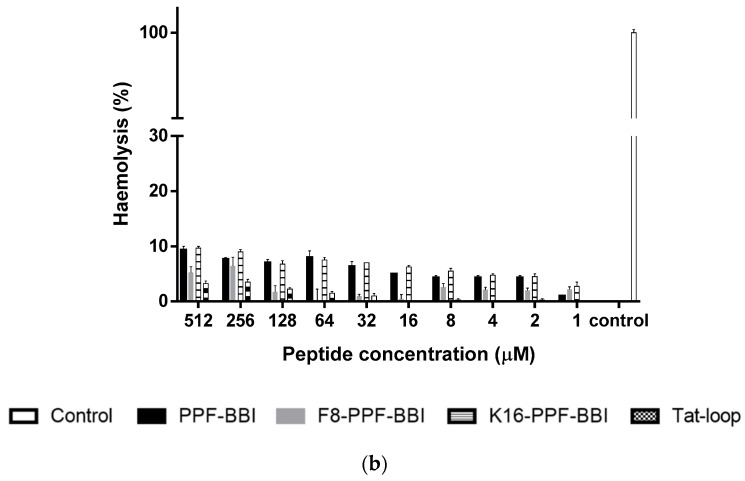
(**a**) The cell viability of the cancer cell lines H460, H157, H23, H838, HT-29, PC-3, U251MG and HMEC-1 at 4 mM 5-FU (stripe bar), 100 µM and 10 µM PPF-BBI, F^8^-PPF-BBI, K^16^-PPF-BBI and Tat-loop. The control represents the cell viability without any treatments. The statistical significance of difference was analyzed by a one-way ANOVA (* *p* < 0.05, **** *p* < 0.0001). (**b**) The haemolysis rates of PPF-BBI, F^8^-PPF-BBI, K^16^-PPF-BBI and Tat-loop on erythrocytes after being incubated for 4 h. The incubation of erythrocytes with 2% (*v*/*v*) Triton X-100 was designated as a positive control (100% haemolysis).

PPF-BBI and its analogues were subjected to an MTT assay using a series of lung cancer cell lines (H460, H157, H23 and H838) and other cancer cell lines (HT29, PC-3, U251MG), and were also tested on a human normal cell (HMEC-1) (Figure 4a). Among these, only Tat-loop inhibited the growth of H460 and H157 at a concentration of 100 µM. All of the peptides showed a slight inhibition on HMEC-1. Furthermore, they also exhibited a low degree of haemolytic activity on horse erythrocytes (Figure 4b).

## 4. Discussion

In this study, we report the identification and bioactivity evaluations of PPF-BBI, a novel Bowman-Birk type protease inhibitor from the skin secretion of the Fukien gold-striped pond frog, *Pelophylax plancyi fukienensis*. In addition, we also examine the bioactivity of three rationally designed analogues of PPF-BBI, F^9^-PPF-BBI, K^16^-PPF-BBI and Tat-loop.

Like other BBI-type peptides, PPF-BBI has potent protease inhibitory activities. The specificity of inhibition is determined by whether the P_1_ position residue can fit into the S1 pocket of protease. Based on previous reports, Lys as the P_1_ position is optimal for trypsin inhibition, and Phe is optimal for chymotrypsin [11,27]. Similarly, PPF-BBI, which has a Lys at the P1 site, showed a strong trypsin inhibitory activity and substitution of the Lys at the P_1_ position, with Phe leading to the elimination of the trypsin inhibition and giving rise to a chymotrypsin inhibitory activity. In the tryptase activity assay, PPF-BBI displayed a mild potency toward tryptase with a Ki value of 30.52 µM. K^16^-PPF-BBI shows a three-fold better inhibition with a Ki value of 9.67 µM, but intriguingly, Tat-loop and TIL lost their inhibitory activity against tryptase, even though they retained their trypsin inhibitory activity. Indeed, this contradicts a previous study [28], which demonstrated that short BBI-derived cyclic peptides had an inhibitory activity against tryptase, even though tryptase is seen as unique due to its resistance to all known endogenous proteinase inhibitors [29]. As the data of K^16^-PPF-BBI showed here, the substitution of Lys at the C-terminus improved the tryptase inhibitory activity, indicating that the C-terminal extensive residue might contribute to the binding between the BBI peptide and tryptase. Therefore, the lack of such an extension of TIL and Tat-loop could eliminate the affinity to the reactive pocket of tryptase, so that both cannot produce any inhibitory activity.

The mode of action of Ranacyclins is different from most known positively charged antimicrobial peptides. They bind and insert into both zwitterionic and negatively charged membranes, and they presumably form transmembrane pores without bacteria wall damage. Indeed, it has been reported that Ranacyclins E and T have a great potential as antimicrobials [30]. PPF-BBI and K^16^-PPF-BBI shared a high sequence similarity with Ranacyclin members, and they were also found to have moderate effects on the tested microorganisms, as was expected. However, K^16^-PPF-BBI was shown to have a better effect, which is possibly because one more lysine increases its positive charges, and it is easier to get it close to the negative groups of the cell membrane.

Tat, a cationic-rich cell-penetrating peptide derived from the HIV protein, has been used to conjugate with other compounds to enhance the cell penetrating activity. In the previous study, the design of Tat-fusion biopeptides demonstrated a remarkable improvement on their biological activities [20,21]. In the meantime, the BBI trypsin inhibitory loop was also considered as a drug template that was applied in some studies [14,31]. Therefore, a combination of the Tat peptide with TIL was conducted here, one that could attempt to introduce the cell-penetrating effect and that possessed an inhibition against trypsin-like activity intracellularly.

Interestingly, this is reflected in the significant increase of potency against the tested strains (especially *C. albicans* with MIC of 4 µM) of Tat-loop. However, this does not appear to be due to its impact upon the membrane, as treatment with this peptide does not induce changes in membrane permeability even at high concentrations (4-fold of its MIC versus *C. albicans*). Using the principle of plant protease inhibitors, it would be pertinent to evaluate the antifungal activity of Tat-loop, possibly because it might interfere with the trypsin-mediated activation of the chitin synthase zymogen and further affect the process of cell wall development [32].

In the report by Zhang et al. [12], a Bowman-Birk type chymotrypsin inhibitory peptide, HECI, and its analogue, K^9^-HECI, exhibited great anti-proliferative potency against H157, PC-3 and MCF-7. Also, PE-BBI was reported to have an inhibition effect on several colon cancer cell lines [13]. In our study, Tat-loop suppresses the growth of the lung cancer cells H460 and H157. It was not clear whether Tat-loop could enter the nucleus and bind the target receptor in vitro; compared to the native peptide, Tat-loop exhibited a slight improvement of the anti-proliferative activity. Moreover, a low haemolytic activity was observed when Tat-loop was assessed, which further supports the data which indicates that it does not exhibit cytotoxicity. Similarly, no significant haemolytic activity was observed with the parental peptide and the other synthetic analogues. This indicates that the Tat-loop will not have toxicity issues and lays a solid foundation for further in vivo research.

In addition, PPF-BBI, F^9^-PPF-BBI and K^16^-PPF-BBI initiated a slight conversion from a random coil into a β-structure in the TFE buffer due to assisting the folding of the secondary structure [33]. However, Tat-loop did not show the band shift to 210 nm, but around 197 nm, which indicated that it mainly formed a random coil in both aqueous and TFE buffer [26]. We assume that BBI-related peptides might exhibit a certain degree of β-sheet structure involved in binding with enzyme; therefore, Tat-loop demonstrated a lower inhibitory activity against trypsin compared with the others. Although the spectra data showed slightly different results, the major part of the secondary structure of the BBI-related peptides is random coil and β-sheet, which is consistent with previous studies [11,25,34].

## 5. Conclusions

In summary, PPF-BBI is a naturally occurring peptide with a remarkable trypsin and tryptase inhibitory activity, as well as a moderate antimicrobial activity. This provided the basis for the rational design of further multifunctional protease inhibitors. Moreover, this is the first report investigating the addition of a cell-penetrating peptide to an amphibian skin-derived protease inhibitor. Tat-loop has high potency against *C. albicans*, which also results from its inhibition of trypsin, which might have potential towards fungal diseases. However, the mechanism by which Tat-loop impacts upon *C. albicans* is unclear, and further research is required to determine how this peptide exerts its impact upon this fungus.

## Figures and Tables

**Table 1:** The sequence and positive charge of *Pelophlax plancyi fukienesis* Bowman-Birk-type inhibitor (PPF-BBI) and its rational design analogues.

Peptide Name	Sequence	Positive Charge
PPF-BBI	ALRGCWT**K**SIPPKPCP-amide	+4
F^8^-PPF-BBI	ALRGCWT**K**SIPPKPCP-amide	+3
K^16^-PPF-BBI	ALRGCWT**K**SIPPKPC**K**-amide	+5
Tat-loop	RKKRRQRRRCWT**K**SIPPKPC	+10

The highly conserved loop is shaded, and the substituted sites are in bold.

**Table 2:** PPF-BBI and its analogues against trypsin, chymotrypsin and tryptase.

Peptide	Name	Ki (µM) of Trypsin	Ki (µM) of Tryptase	Ki (µM) of Chymotrypsin
ALRGCWT**K**SIPPKPCP-amide	PPF-BBI	0.17	30.73	N.I.*
ALRGCWT**K**SIPPKPCP-amide	F^8^-PPF-BBI	N.I.*	N.I.*	0.85
ALRGCWT**K**SIPPKPC**K**-amide	K^16^-PPF-BBI	0.112	9.67	N.I.*
RKKRRQRRRCWT**K**SIPPKPC	Tat-loop	0.607	N.I.*	N.I.*
CWT**K**SIPPKPC	TIL	0.741	N.I.*	N.I.*

The highly conserved loop is shaded, and the substituted sites are in bold. N.I.* means that no inhibition was observed.

**Table 3:** The minimal inhibitory concentrations (µM) and minimal bactericide concentrations (µM) of PPF-BBI and the synthetic analogue peptides against microorganisms.

Microorganisms	MIC/MBC (µM)					
	PPF-BBI	K^16^-PPF-BBI	F^8^-PPF-BBI	Tat	Tat-loop	TIL
*S. aureus*	128/128	64/64	>512	512/512	128/128	>512
*E. coli*	128/128	128/128	>512	256/256	128/128	>512
*C. albicans*	512/512	128/128	>512	>512	4/8	>512
MRSA	>512	512/512	>512	>512	256/512	>512
*P. aeruginosa*	>512	512/512	>512	>512	256/256	>512

## Supplementary Materials

The following are available online at https://www.mdpi.com/2218-273X/9/7/280/s1, Figure S1: The nucleotide and translated open-reading frame amino acid sequence of cloned cDNA encoding the biosynthetic precursor of PPF-BBI from a skin secretion of *Pelophylax plancyi fukienensis*. Figure S2. Region of reverse-phase HPLC chromatogram of the skin secretions of *Pelopholax plancyi fukienesis*. Figure S3. The identification of PPF-BBI from the skin secretion of *Pelopholax plancyi fukienesis*. Figure S4. The identification of other peptides used in this study. Figure S5. The inhibitory activity of PPF-BBI and the analogues on trypsin, chymotrypsin and tryptase.
