# Supplementary material for: Discovery and Rational Design of a Novel Bowman-Birk Related Protease Inhibitor
Source: Biomolecules. 2019 Jul 14;9(7):280. doi: 10.3390/biom9070280 (PMC6681222; doi:10.3390/biom9070280)
Supplement: Supplementary file 1 [file biomolecules-09-00280-s001.pdf]

biomolecules

Discovery and rational design of a novel Bowman-Birk related protease inhibitor

Supplementary materials

```
       M  F  T  M   K  K  S   L  L  F   L  F  F  L   G  T  I ·
  1 ATGTTCACCA TGAAGAAATC CCTGTTATTC CTTTTCTTTC TTGGGACCAT
    TACAAGTGGT ACTTCTTTAG GGACAATAAG GAAAAGAAAG AACCCTGGTA
    · N  F  S   L  C  E  Q   E  R  D   A  D  E   E  D  G  R ·
 51 CAACTTTTCT CTCTGTGAGC AAGAGAGAGA TGCCGATGAA GAAGATGGAA
    GTTGAAAAGA GAGACACTCG TTCTCTCTCT ACGGCTACTT CTTCTACCTT
    ·  D  E  P   E  E  R   D  V  E  V   K  R  A   L  R  G
101 GAGACGAGCC AGAGGAAAGA GATGTCGAAG TAAAAAGAGC ACTCAGAGGG
    CTCTGCTCGG TCTCCTTTCT CTACAGCTTC ATTTTTCTCG TGAGTCTCCC
     C  W  T  K   S  I  P   P  K  P   C  P  G  K   R  *
151 TGTTGGACCA AGAGTATACC ACCTAAGCCT TGTCCAGGAA AAAGATAAAA
    ACAACCTGGT TCTCATATGG TGGATTCGGA ACAGGTCCTT TTTCTATTTT
201 CTCAAATTGG AAGTCATCTA ATTTGGAATA TCATTTAGCT AAATGCTATG
    GAGTTTAACC TTCAGTAGAT TAAACCTTAT AGTAAATCGA TTTACGATAC
251 TCAGATTAAA AATTAAATAA CTAAATACTA TCATATACAG AAAAAAAAAA
    AGTCTAATTT TTAATTTATT GATTTATGAT AGTATATGTC TTTTTTTTTT
301 AAAAAAAAAA AAAA
    TTTTTTTTTT TTTT
```

Figure S1. The nucleotide and translated open-reading frame amino acid sequence of cloned cDNA encoding the biosynthetic precursor of PPF-BBI from a skin secretion of *Pelophylax plancyi fukienensis*. The putative signal peptide is double-underlined, mature peptide is single-underlined and the stop codon is indicated by an asterisk.

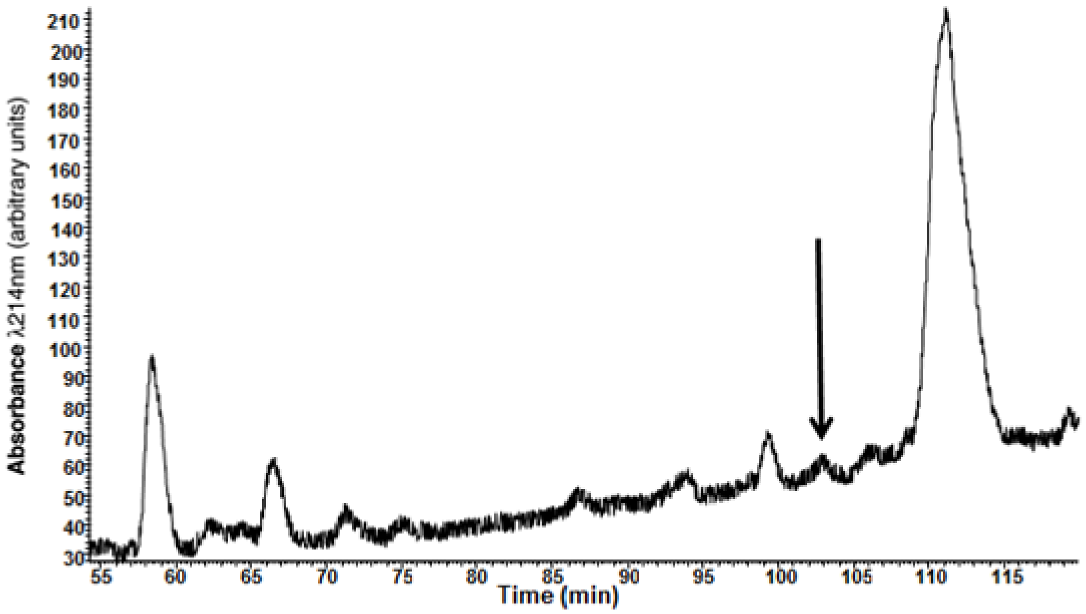

Figure S2. Region of reverse-phase HPLC chromatogram of the skin secretions of *Pelopholax plancyi fukienesis*. The elution position/retention time of PPF-BBI is indicated (arrow). Column: Jupiter C-5, 5-μm particle, 300 Å pore, 250 × 10 mm (Phenomenex, UK). Elution condition: linear gradient formed from 0.05/99.95 (v/v) TFA/water to 0.05/19.95/80.00 (v/v/v) TFA/water/acetonitrile over 240 min at a flow rate of 1 ml/min.

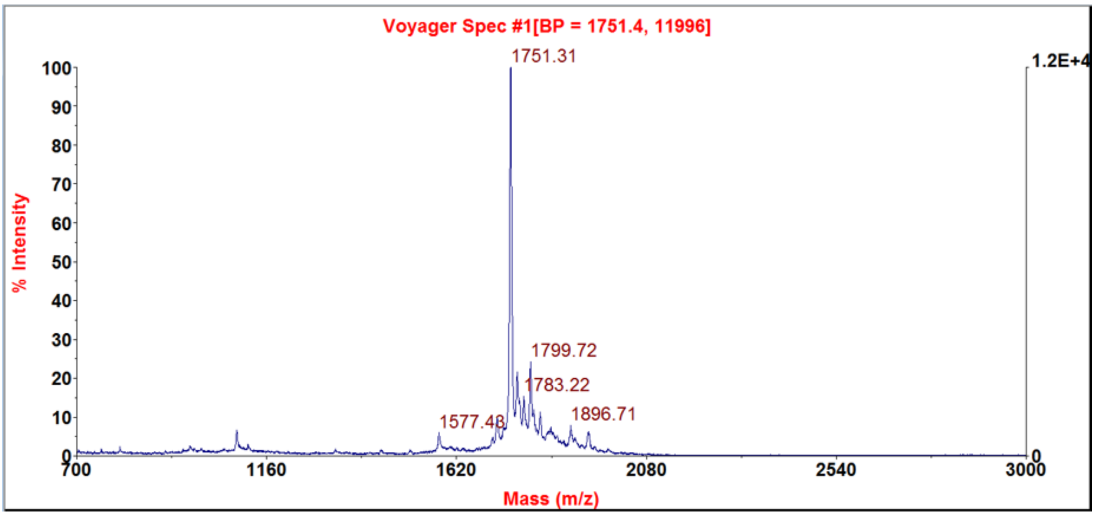

A

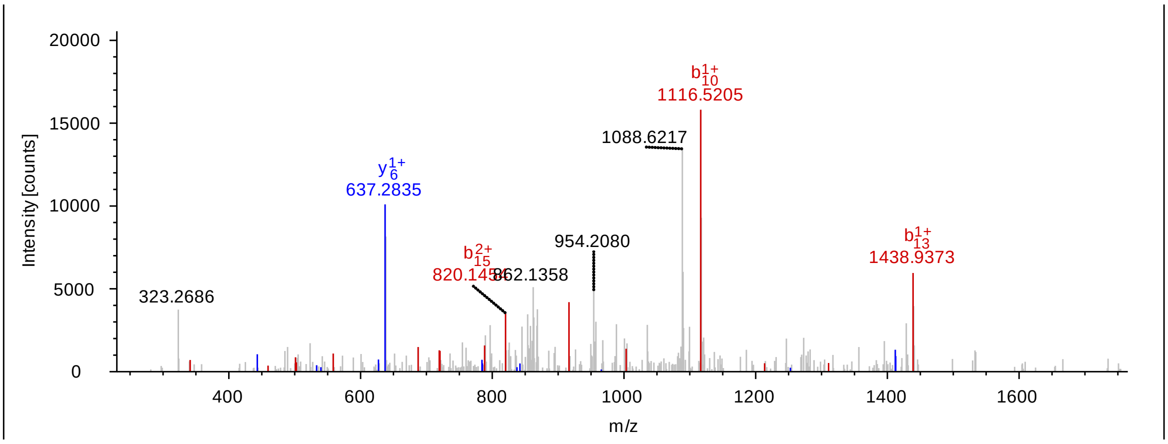

B

#1	b(1+)	b(2+)	Seq.	y(1+)	y(2+)	#2
1	72.04440	36.52584	A			16
2	185.12847	93.06787	L	1681.90299	841.45513	15
3	341.22959	171.11843	R	1568.81892	784.91310	14
4	398.25106	199.62917	G	1412.71780	706.86254	13
5	501.26025	251.13376	C	1355.69633	678.35180	12
6	687.33957	344.17342	W	1252.68714	626.84721	11
7	788.38725	394.69726	T	1066.60782	533.80755	10
8	916.48222	458.74475	K	965.56014	483.28371	9
9	1003.51425	502.26076	S	837.46517	419.23622	8
10	1116.59832	558.80280	I	750.43314	375.72021	7
11	1213.65109	607.32918	P	637.34907	319.17817	6
12	1310.70386	655.85557	P	540.29630	270.65179	5
13	1438.79883	719.90305	K	443.24353	222.12540	4
14	1535.85160	768.42944	P	315.14856	158.07792	3
15	1638.86079	819.93403	C	218.09579	109.55153	2
16			P-Amidated	115.08660	58.04694	1

C

Figure S3. The identification of PPF-BBI from the skin secretion of *Pelophylax plancyi fukienesis*. (A) The mass trace of the fraction indicated in Figure S2. (B) The MS/MS spectrum of doubly-charged precursor ion of PPF-BBI in the fraction. (C) Predicted b-ion and y-ion MS/MS fragment ion series (singly- and doubly-charged) of PPF-BBI. The actual ions detected in MS/MS spectra are coloured red and blue.

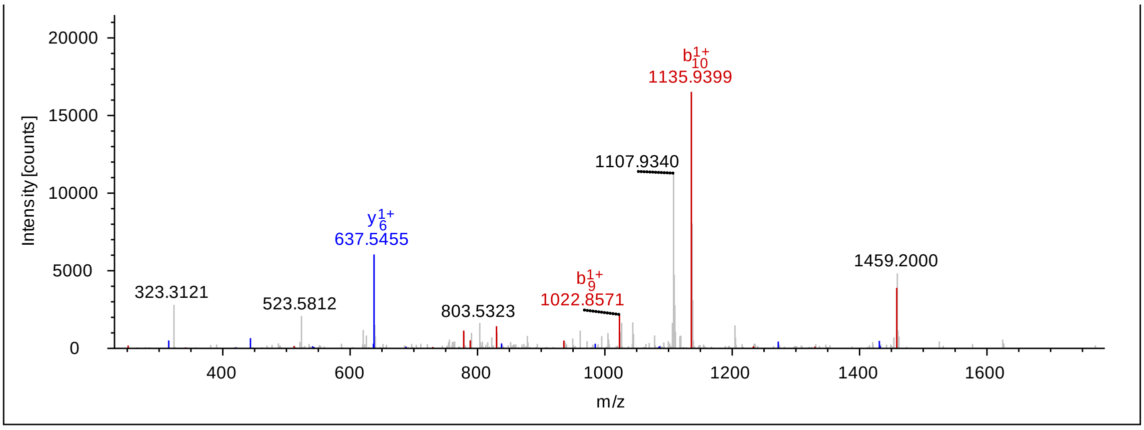

#1	b(1+)	b(2+)	Seq.	y(1+)	y(2+)	#2
1	72.04440	36.52584	A			16
2	185.12847	93.06787	L	1700.87644	850.94186	15
3	341.22959	171.11843	R	1587.79237	794.39982	14
4	398.25106	199.62917	G	1431.69125	716.34926	13
5	501.26025	251.13376	C	1374.66978	687.83853	12
6	687.33957	344.17342	W	1271.66059	636.33393	11
7	788.38725	394.69726	T	1085.58127	543.29427	10
8	935.45567	468.23147	F	984.53359	492.77043	9
9	1022.48770	511.74749	S	837.46517	419.23622	8
10	1135.57177	568.28952	I	750.43314	375.72021	7
11	1232.62454	616.81591	P	637.34907	319.17817	6
12	1329.67731	665.34229	P	540.29630	270.65179	5
13	1457.77228	729.38978	K	443.24353	222.12540	4
14	1554.82505	777.91616	P	315.14856	158.07792	3
15	1657.83424	829.42076	C	218.09579	109.55153	2
16			P-Amidated	115.08660	58.04694	1

(A)

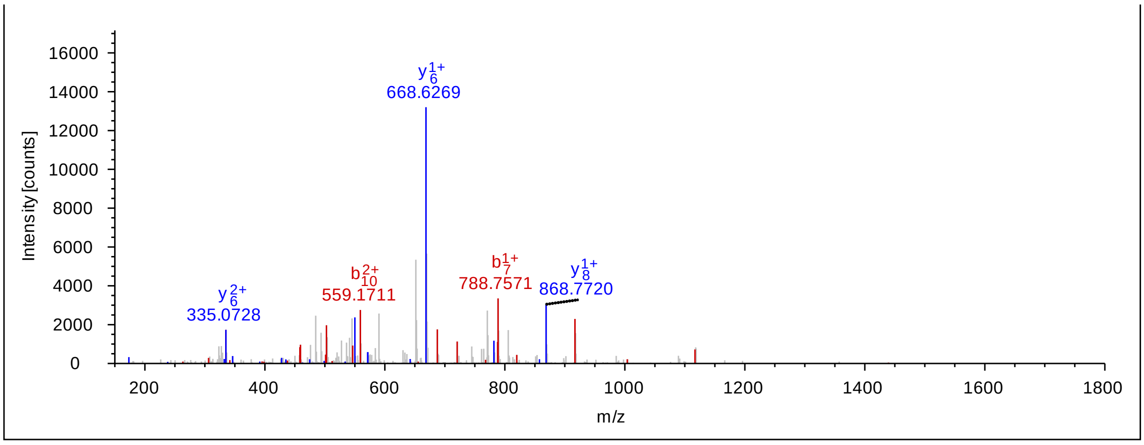

#1	b(1+)	b(2+)	b(3+)	Seq.	y(1+)	y(2+)	y(3+)	#2
1	72.04440	36.52584	24.68632	A				16
2	185.12847	93.06787	62.38101	L	1712.94519	856.97623	571.65325	15
3	341.22959	171.11843	114.41471	R	1599.86112	800.43420	533.95856	14
4	398.25106	199.62917	133.42187	G	1443.76000	722.38364	481.92485	13
5	501.26025	251.13376	167.75827	C	1386.73853	693.87290	462.91769	12
6	687.33957	344.17342	229.78471	W	1283.72934	642.36831	428.58130	11
7	788.38725	394.69726	263.46727	T	1097.65002	549.32865	366.55486	10
8	916.48222	458.74475	306.16559	K	996.60234	498.80481	332.87230	9
9	1003.51425	502.26076	335.17627	S	868.50737	434.75732	290.17397	8
10	1116.59832	558.80280	372.87096	I	781.47534	391.24131	261.16330	7
11	1213.65109	607.32918	405.22188	P	668.39127	334.69927	223.46861	6
12	1310.70386	655.85557	437.57280	P	571.33850	286.17289	191.11768	5
13	1438.79883	719.90305	480.27113	K	474.28573	237.64650	158.76676	4
14	1535.85160	768.42944	512.62205	P	346.19076	173.59902	116.06844	3
15	1638.86079	819.93403	546.95845	C	249.13799	125.07263	83.71751	2
16				K-Amidated	146.12880	73.56804	49.38112	1

(B)

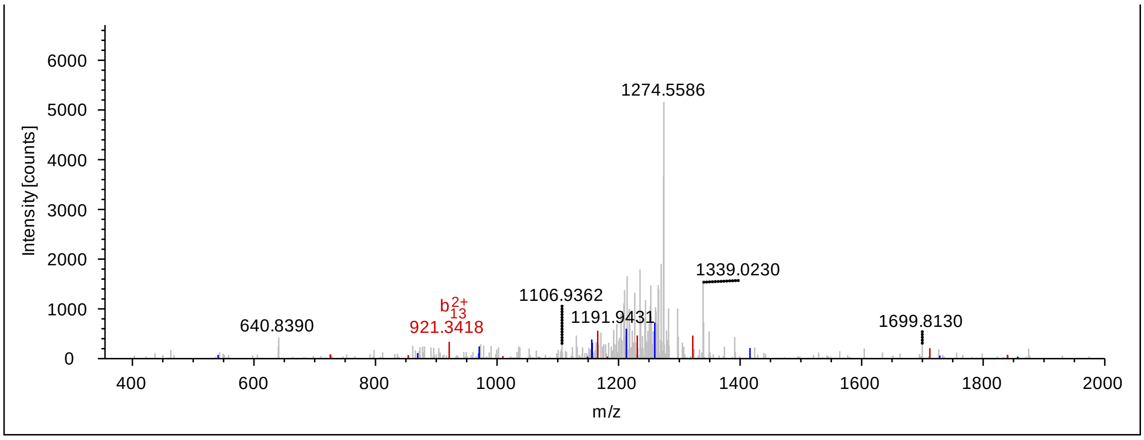

#1	b(1+)	b(2+)	Seq.	y(1+)	y(2+)	#2
1	157.10840	79.05784	R			20
2	285.20337	143.10532	K	2424.38169	1212.69448	19
3	413.29834	207.15281	K	2296.28672	1148.64700	18
4	569.39946	285.20337	R	2168.19175	1084.59951	17
5	725.50058	363.25393	R	2012.09063	1006.54895	16
6	853.55916	427.28322	Q	1855.98951	928.49839	15
7	1009.66028	505.33378	R	1727.93093	864.46910	14
8	1165.76140	583.38434	R	1571.82981	786.41854	13
9	1321.86252	661.43490	R	1415.72869	708.36798	12
10	1424.87171	712.93949	C	1259.62757	630.31742	11
11	1610.95103	805.97915	W	1156.61838	578.81283	10
12	1711.99871	856.50299	T	970.53906	485.77317	9
13	1840.09368	920.55048	K	869.49138	435.24933	8
14	1927.12571	964.06649	S	741.39641	371.20184	7
15	2040.20978	1020.60853	I	654.36438	327.68583	6
16	2137.26255	1069.13491	P	541.28031	271.14379	5
17	2234.31532	1117.66130	P	444.22754	222.61741	4
18	2362.41029	1181.70878	K	347.17477	174.09102	3
19	2459.46306	1230.23517	P	219.07980	110.04354	2
20			C	122.02703	61.51715	1

(C)

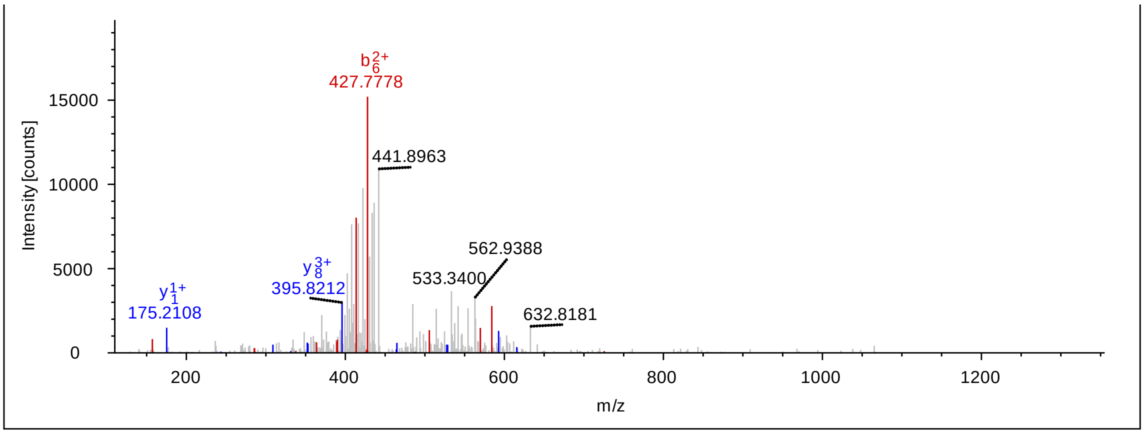

#1	b(1+)	b(2+)	b(3+)	Seq.	y(1+)	y(2+)	y(3+)	#2
1	157.10840	79.05784	53.04098	R				9
2	285.20337	143.10532	95.73931	K	1183.77196	592.38962	395.26217	8
3	413.29834	207.15281	138.43763	K	1055.67699	528.34213	352.56385	7
4	569.39946	285.20337	190.47134	R	927.58202	464.29465	309.86552	6
5	725.50058	363.25393	242.50504	R	771.48090	386.24409	257.83182	5
6	853.55916	427.28322	285.19124	Q	615.37978	308.19353	205.79811	4
7	1009.66028	505.33378	337.22494	R	487.32120	244.16424	163.11192	3
8	1165.76140	583.38434	389.25865	R	331.22008	166.11368	111.07821	2
9				R	175.11896	88.06312	59.04450	1

(D)

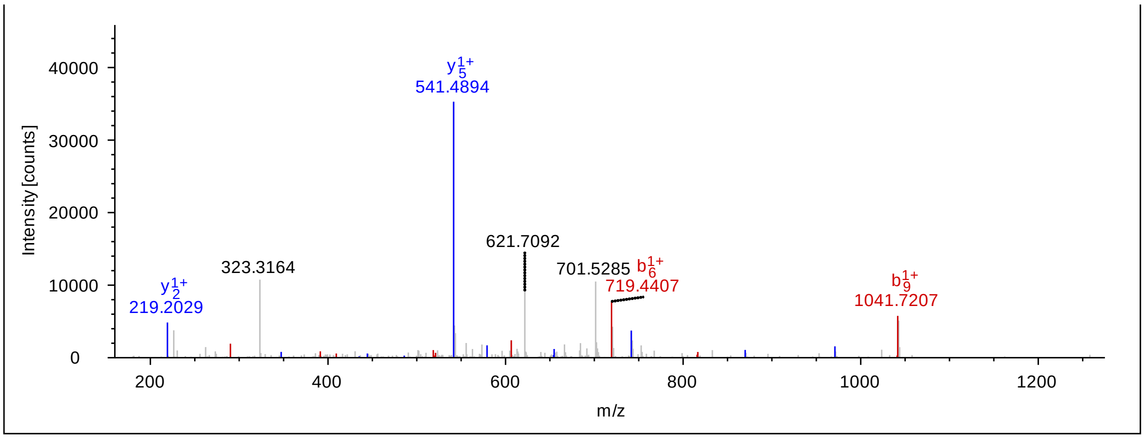

#1	b(1+)	b(2+)	Seq.	y(1+)	y(2+)	#2
1	104.01647	52.51187	C			11
2	290.09579	145.55153	W	1156.61838	578.81283	10
3	391.14347	196.07537	T	970.53906	485.77317	9
4	519.23844	260.12286	K	869.49138	435.24933	8
5	606.27047	303.63887	S	741.39641	371.20184	7
6	719.35454	360.18091	I	654.36438	327.68583	6
7	816.40731	408.70729	P	541.28031	271.14379	5
8	913.46008	457.23368	P	444.22754	222.61741	4
9	1041.55505	521.28116	K	347.17477	174.09102	3
10	1138.60782	569.80755	P	219.07980	110.04354	2
11			C	122.02703	61.51715	1

(E)

Figure S4. The identification of other peptides used in this study. (A) F[8]-PPF-BBI (B) K[16]-PPF-BBI (C) Tat-loop (D) Tat (E) TIL

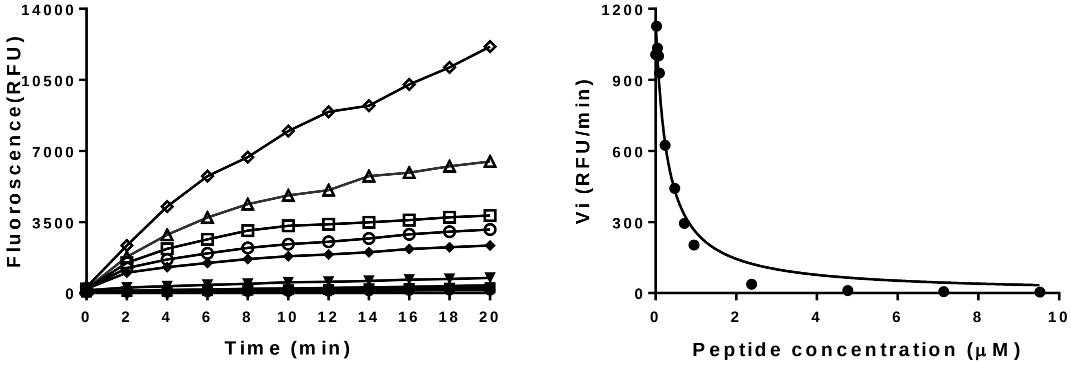

Fluoroscence(RFU)
Time (min)
Vi (RFU/min)
Peptide concentration (μM)

(a)

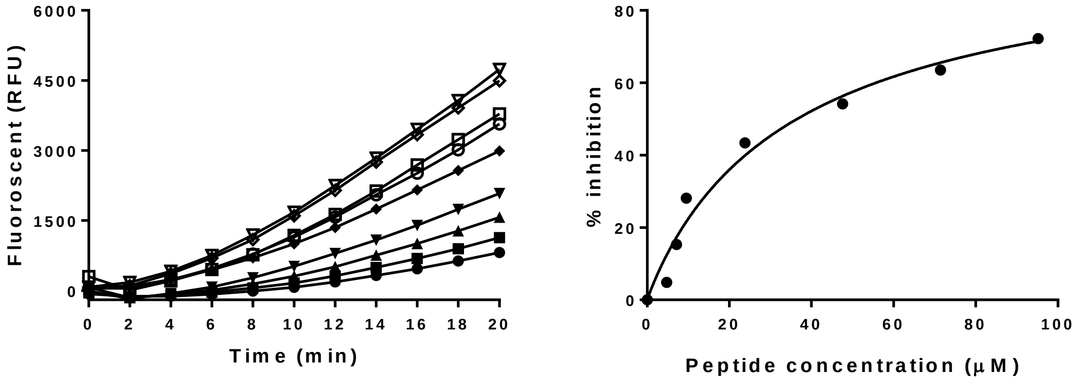

Fluoroscent (RFU)
Time (min)
% inhibition
Peptide concentration (μM)

(b)

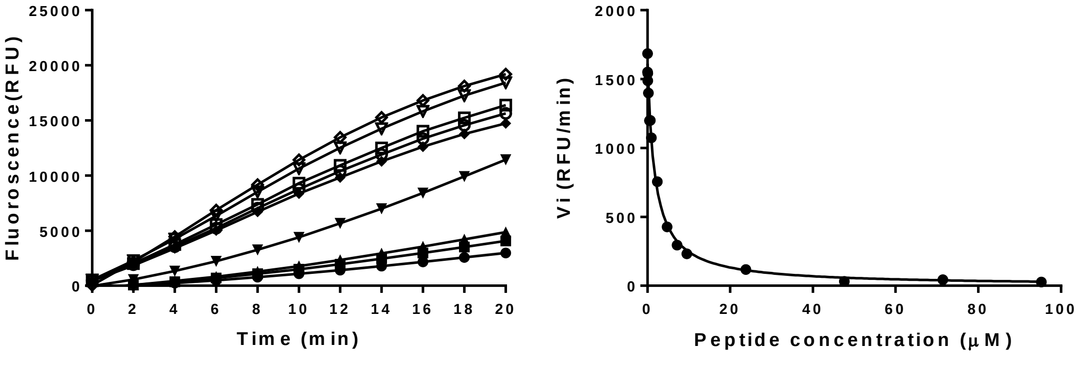

Fluoroscence(RFU)
Time (min)
Vi (RFU/min)
Peptide concentration (μM)

(c)

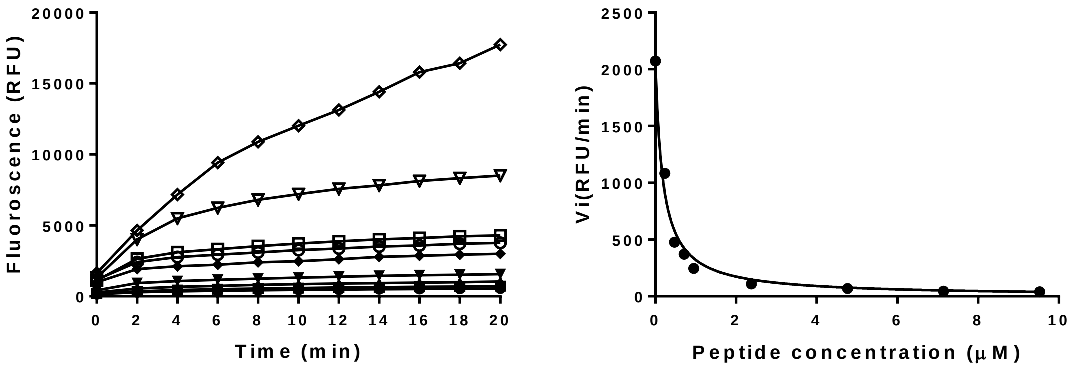
Fluoroscence (RFU)
20000
15000
10000
5000
0
0 2 4 6 8 10 12 14 16 18 20
Time (min)
Vi(RFU/min)
2500
2000
1500
1000
500
0
0 2 4 6 8 10
Peptide concentration (µM)

(d)

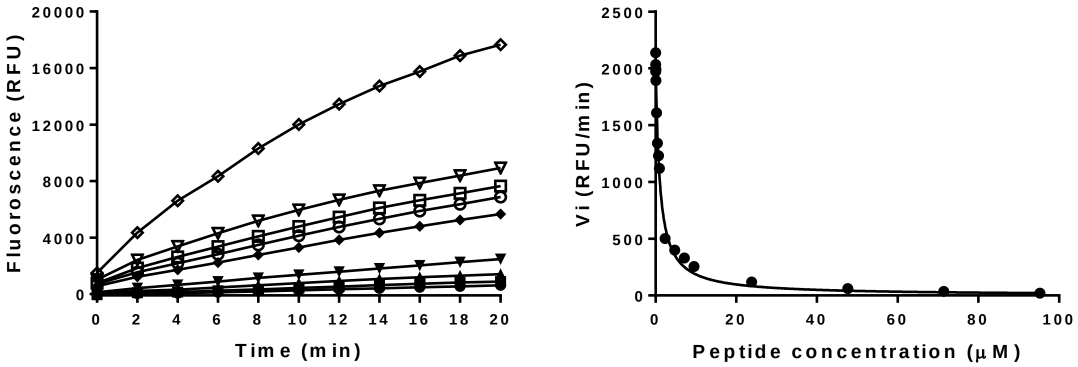
Fluoroscence (RFU)
20000
16000
12000
8000
4000
0
0 2 4 6 8 10 12 14 16 18 20
Time (min)
Vi (RFU/min)
2500
2000
1500
1000
500
0
0 20 40 60 80 100
Peptide concentration (µM)

(e)

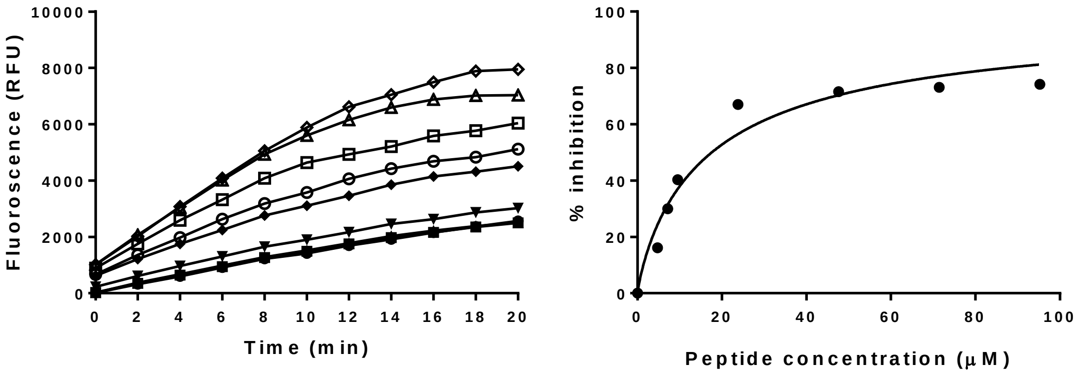
Fluoroscence (RFU)
10000
8000
6000
4000
2000
0
0 2 4 6 8 10 12 14 16 18 20
Time (min)
% inhibition
100
80
60
40
20
0
0 20 40 60 80 100
Peptide concentration (µM)

(f)

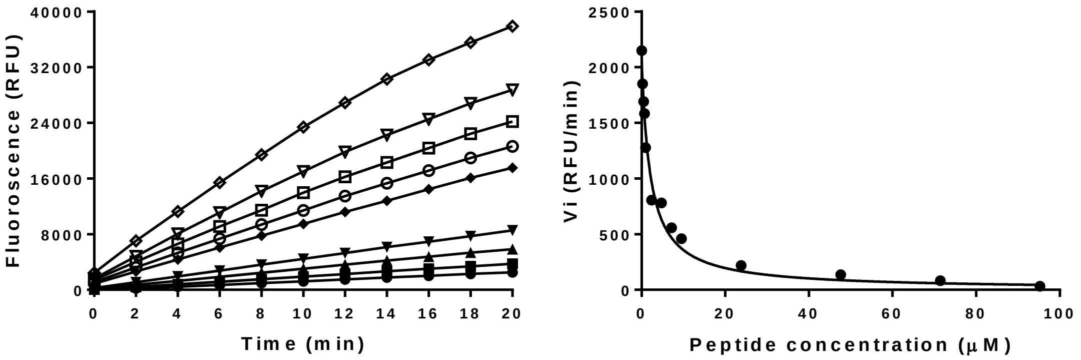

(g)

Figure S5. Inhibitory activity of PPF-BBI and its analogues on trypsin, chymotrypsin and tryptase. Substrate with no treatment of peptide (0 μM) was used as the negative control. (a) Progress curves and corresponding Morrison plot for trypsin proteolysis in the presence of different concentrations of PPF-BBI. (b) Progress curves and corresponding Morrison plot for tryptase proteolysis in the presence of different concentrations of PPF-BBI. (c) Progress curves and corresponding Morrison plot for chymotrypsin proteolysis in the presence of different concentrations of F[8]-PPF-BBI. (d) Progress curves and corresponding Morrison plot for trypsin proteolysis in the presence of different concentrations of K[16]-PPF-BBI. (e) Progress curves and corresponding Morrison plot for trypsin proteolysis in the presence of different concentrations of Tat-loop. (f) Progress curves and corresponding Morrison plot for tryptase proteolysis in the presence of different concentrations of K[16]-PPF-BBI. (g) Progress curves and corresponding Morrison plot for trypsin proteolysis in the presence of different concentrations of TIL (● 95.2 μM, ■ 71.4 μM, ▲ 47.6 μM, ▼ 23.8 μM, ◆ 9.52 μM, ○ 7.14 μM, □ 4.76μM, △ 2.38 μM, ◇ 0 μM).
